# Supplementary material for: Two subtypes of GTPase-activating proteins coordinate tip growth and cell size regulation in Physcomitrium patens
Source: Nat Commun. 2023 Nov 4;14:7084. doi: 10.1038/s41467-023-42879-y (PMC10625565; doi:10.1038/s41467-023-42879-y)
Supplement: Supplementary file 1 — Supplementary Information [file 41467_2023_42879_MOESM1_ESM.pdf]

Supplementary Information

Two subtypes of GTPase-activating proteins coordinate tip growth and cell size regulation in *Physcomitrium patens*

Jingtong Ruan[1,3], Linyu Lai[1,3], Hongxin Ou[1,2], Peishan Yi[1,4]

[1] Key Laboratory of Bio-Resource and Eco-Environment of Ministry of Education, College of Life Sciences, Sichuan University, No. 24 South Section 1, Yihuan Road, Wuhou District, Chengdu, Sichuan 610064, P. R. China.

[2] Present address: School of Life Sciences, Tsinghua University, Beijing, 100084, P. R. China.

[3] These authors contributed equally to this work.

[4] Correspondence: yipeishan@scu.edu.cn

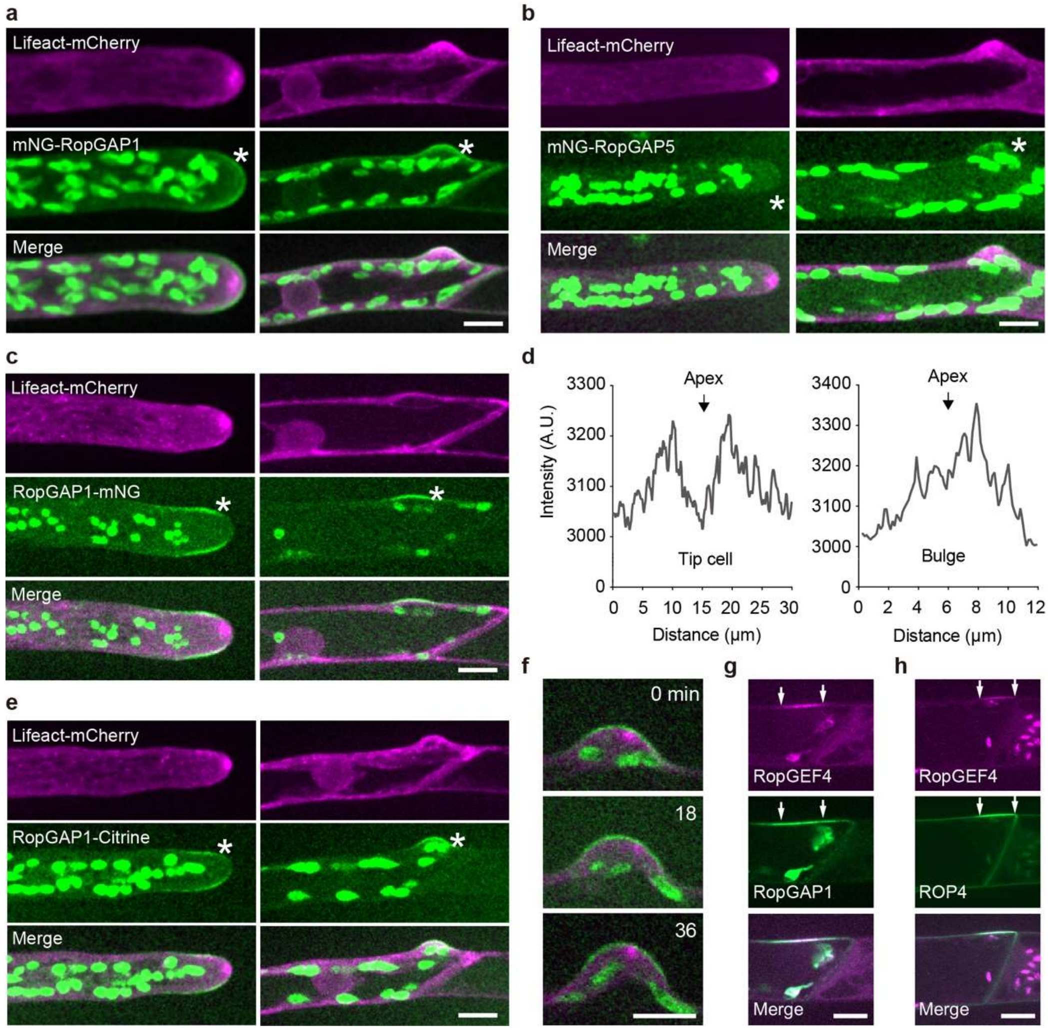

Supplementary Fig. 1: Localization of PpRopGAP1 in tip cells and subapical branching cells. (a) Localization of PpRopGAP1 fused with an N-terminal mNG tag. Actin is labeled with Lifeact-mCherry. Stars indicate the apical membrane of a tip cell or the apical membrane of a bulge in the subapical cell. (b) Localization of PpRopGAP5 fused with an N-terminal mNG tag. (c) Localization of PpRopGAP1 fused with a C-terminal mNG tag. (d) Representative intensity plots of PpRopGAP1-mNG along the apical membrane in a tip cell and at the branching site (bulge) in a subapical cell. Note that the low-intensity area is not present at the branching site. (e) Localization of PpRopGAP1 fused with a C-terminal Citrine tag. (f) Time-lapse images of PpRopGAP1-mNG during bulge growth. Note that the actin foci undergo dynamic assembly and disassembly, while PpRopGAP1-mNG does not exhibit a low-intensity zone at the apex. (g) Colocalization of PpRopGAP1-mNG and PpRopGEF4-mCherry before bulge initiation. (h) Colocalization of PpROP4-mNG and PpRopGEF4-mCherry before bulge initiation. Arrows indicate the boundary of PpRopGEF4 fluorescence. Scale bars in all panels: 10 μm.

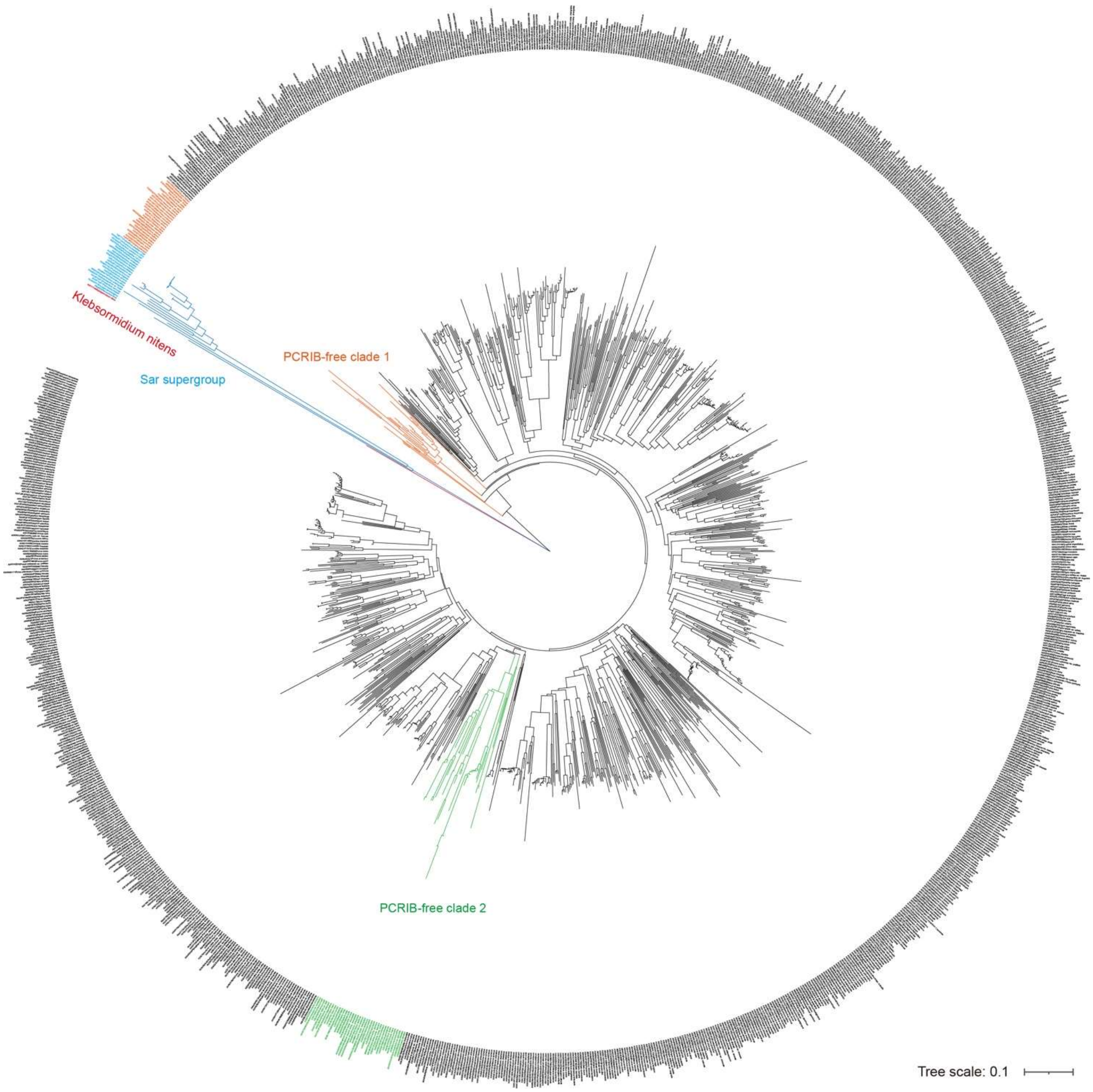

Supplementary Fig. 2: Phylogenetic tree of RopGAPs. In total, 1 244 RopGAPs that comprise a CRIB domain and a RhoGAP domain are retrieved from the InterPro database. Protein sequences are obtained from the Uniprot database and aligned by Clustal Omega. The phylogenetic tree is constructed using the neighboring-joining method with a 1 000 bootstrap value in Mega11. The majority of RopGAPs contain a pre-CRIB (PCRIB) motif preceding the CRIB core sequence. The RopGAP homologs in the Sar outgroup and *Klebsormidium nitens* are indicated in blue and red, respectively. Two small subclades that lack the PCRIB motif are shown in orange and green.

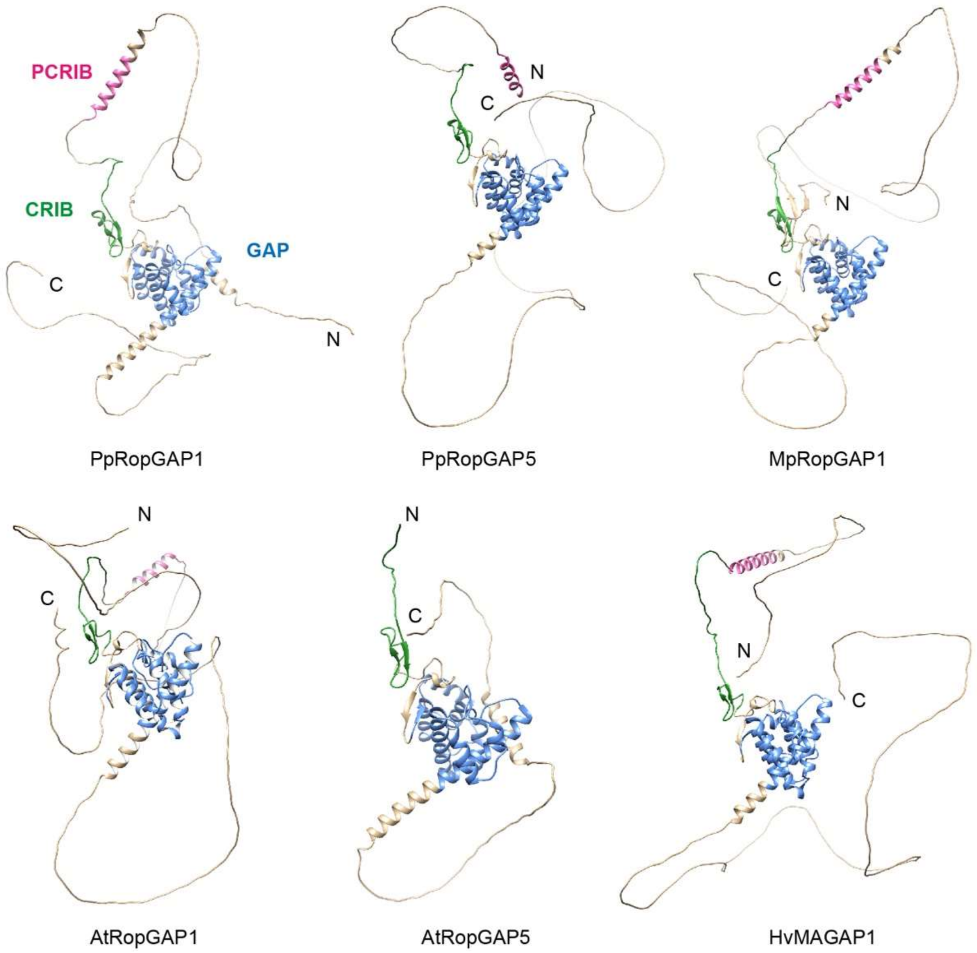

Supplementary Fig. 3: AlphaFold-predicted structures of RopGAPs. The N-terminal (N) and C-terminal (C) ends of each protein are indicated. The PCRIB motif, CRIB motif, and GAP domain are shown in magenta, green, and blue, respectively. Note that the PCRIB motif is predicted to fold into an α-helix, but is absent in AtRopGAP5. Pp, *Physcomitrium patens*; Mp, *Marchantia polymorpha*; At, *Arabidopsis thaliana*; Hv, *Hordeum vulgare*.

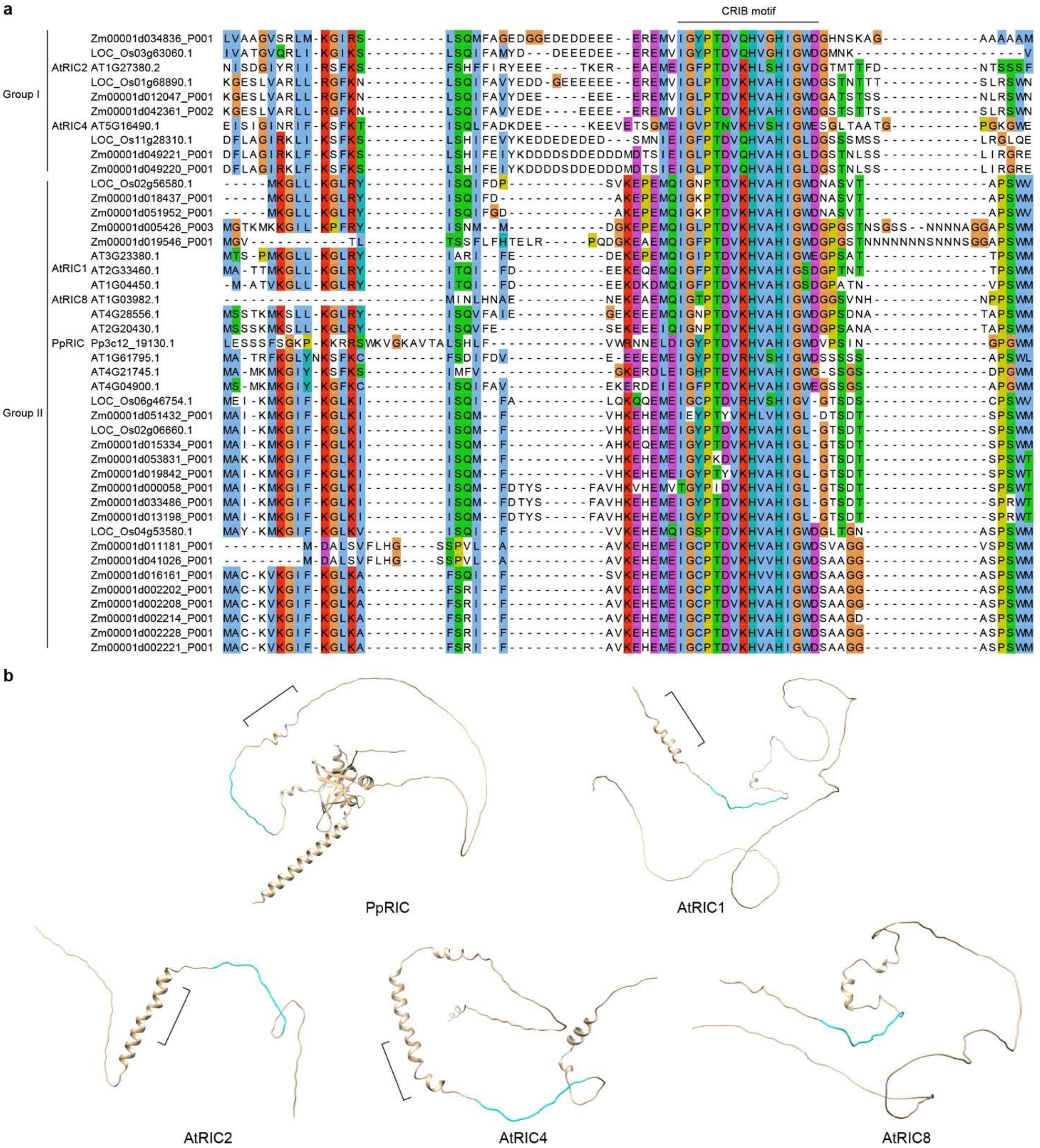

Supplementary Fig. 4: Sequence alignment and predicted structures of RICs. (a) Protein sequences of RICs from *Physcomitrium patens*, Arabidopsis, rice, and maize were aligned using Clustal Omega. Phylogenetically, RICs could be classified into two groups. The CRIB motif as well as its flanking regions is shown. Note that the N-terminal and C-terminal regions are conserved in most RICs. Group I RICs contain extra polyacidic residues immediately before the CRIB motif. (b) Alphafold predicted structures of representative RICs. The conserved region (indicated by brackets) before the CRIB motif (cyan) in RICs is different from those in RopGAPs in sequence. However, it also tends to adopt an α-helical structure except for RICs such as AtRIC8 which lack a full-length N-terminal region.

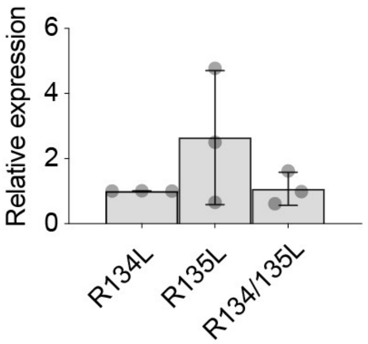

Supplementary Fig. 5: Relative transcriptional expression levels of PpRopGAP1 with single and double mutations in the PCRIB motif. The expression levels were detected by qRT-PCR using the TAF10 as the reference gene. Quantification was performed for three independent biological samples from four technical repeats. The expression of PpRopGAP1(R135L) and PpRopGAP1(R134/135L) was normalized to that of PpRopGAP1(R134L). No significant difference was found for each pair of the data using a one-way ANOVA test.

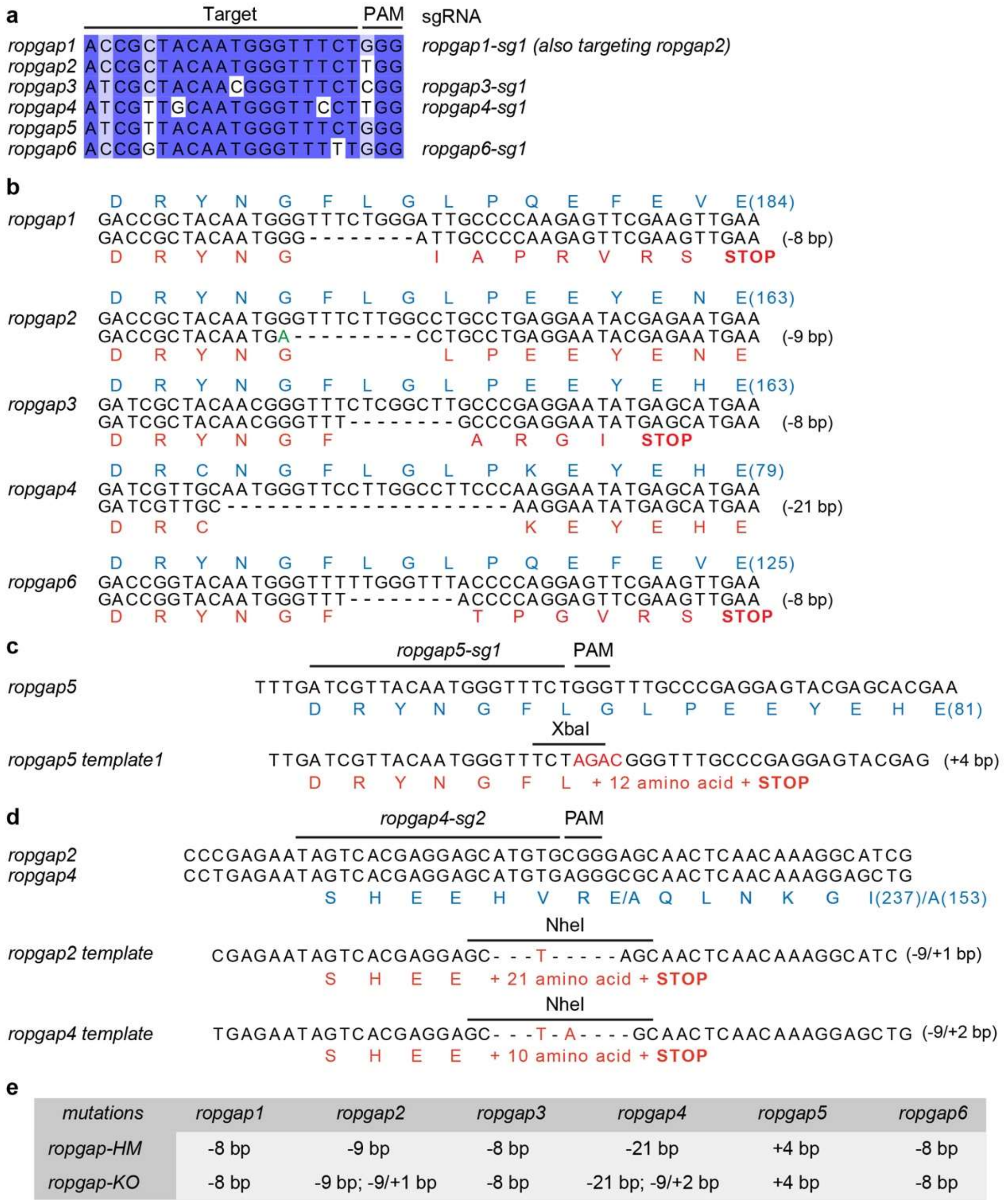

Supplementary Fig. 6: Construction of the *ropgap* mutants. (a) Alignment of CRISPR guide RNA (sgRNA) targets used for the first round of genome editing. Four targets (*ropgap1-sg1*, *ropgap3-sg1*, *ropgap4-sg1*, *ropgap6-sg1*) are cotransformed, of which the *ropgap1-sg1* also targets *ropgap2*. PAM, protospacer adjacent motif. (b) Mutations generated by CRISPR/Cas9 editing. The change of translated amino acids (represented by single letters) is shown below the DNA sequences. (c) Oligonucleotide template-dependent genome editing of *ropgap5*. An *Xba* I site is introduced in the template to facilitate genotyping. (d) Oligonucleotide template-dependent genome editing of *ropgap2* and *ropgap4*. The *ropgap4-sg2* is used for simultaneous editing of *ropgap2* and *ropgap4*. A *Nhe* I site is introduced in each template to facilitate genotyping. (e) Summary of mutations in the final *ropgap* sextuple knockout (KO) or hypomorphic (HM) mutants.

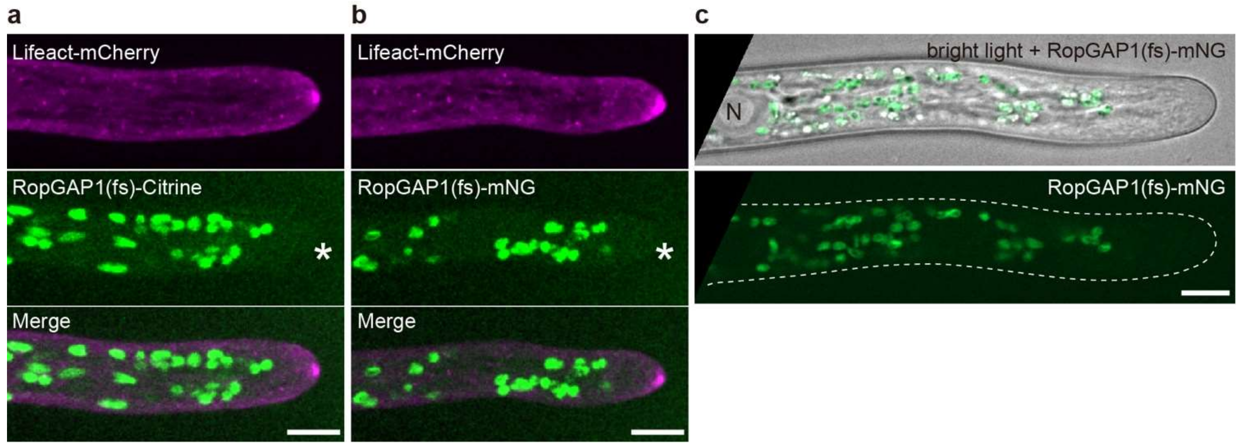

Supplementary Fig. 7: The expression of PpRopGAP1 is completely blocked by frameshift mutations. (a) Expression of the endogenous PpRopGAP1 carrying an 8-bp frameshift mutation (fs) in the CRIB domain and fused with a C-terminal Citrine. No fluorescence is detectable at the apical membrane (star). Actin is labeled with a Lifeact-mCherry reporter. (b) Expression of the endogenous PpRopGAP1 carrying an 8-bp frameshift mutation (fs) in the CRIB domain and fused with a C-terminal mNG. (c) Overexpression of the mutated PpRopGAP1 with a C-terminal mNG driven by EF1α promoter. N, nucleus. Scale bars in all panels: 10 µm.

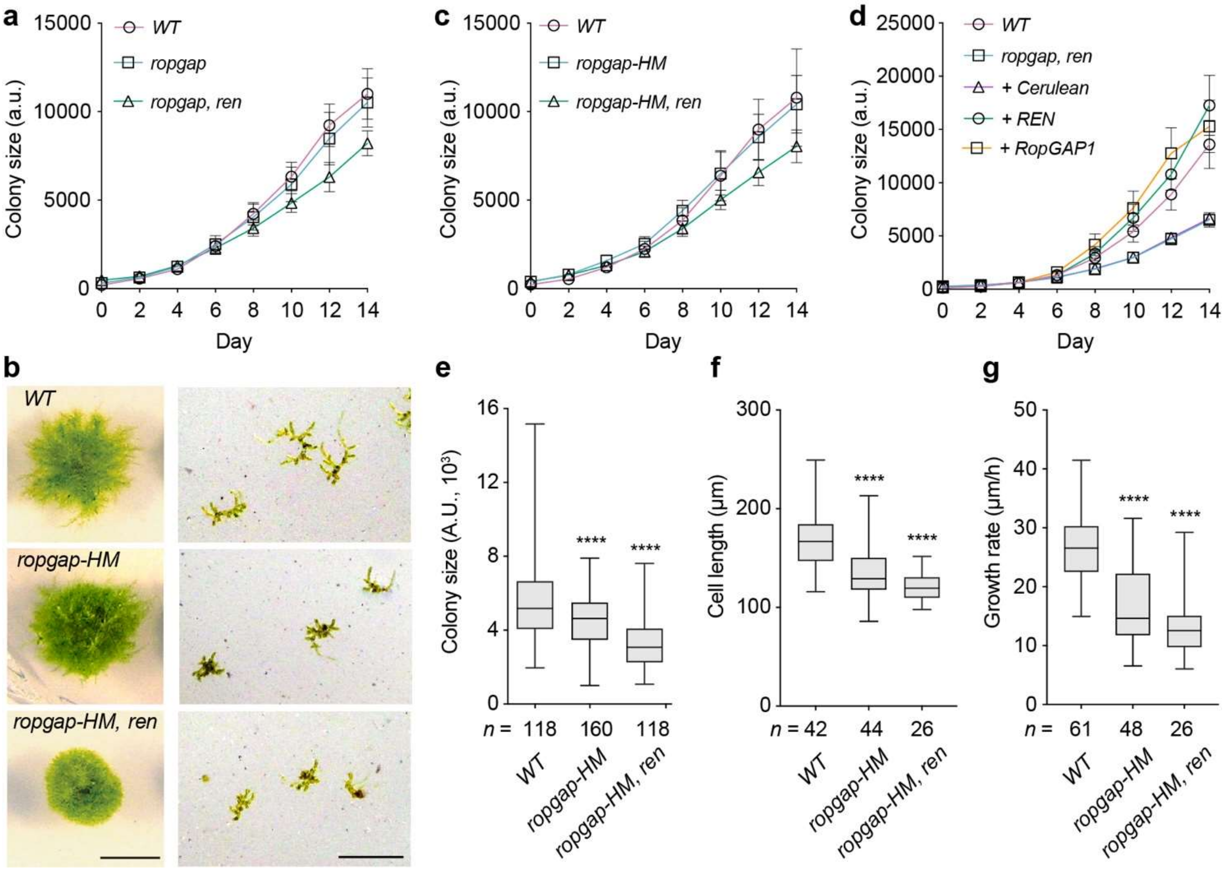

Supplementary Fig. 8: Phenotypes and rescue of PpRopGAP-related mutants. (a) Quantification of the colony size of wild-type (WT) and knockout mutant mosses cultured for two weeks. n = 32 colonies (mean ± SD) for each strain. (b) Colony growth and protoplast regeneration in WT, hypomorphic *ropgap* sextuple mutant (*ropgap-HM*), and *ropgap-HM, ren* septuple mutant. Scale bar: 0.5 cm (left), 0.5 mm (right). (c) Quantification of the colony size of WT and hypomorphic mutant mosses cultured for two weeks. n = 32 colonies (mean ± SD) for each strain. (d) Quantification of the colony size of mutant and rescued mosses cultured for two weeks. n = 16 colonies (mean ± SD) for each strain. (e) Quantification of the colony size of 7-day-old regenerated mosses after protoplasting. (f) Quantification of subapical cell length. (g) Quantification of the growth rate of caulonema tip cells. The number of colonies or cells used for quantification is shown at the bottom in (e-g). Each group is compared with WT and analyzed by a one-way ANOVA test. ****, $p < 0.0001$.

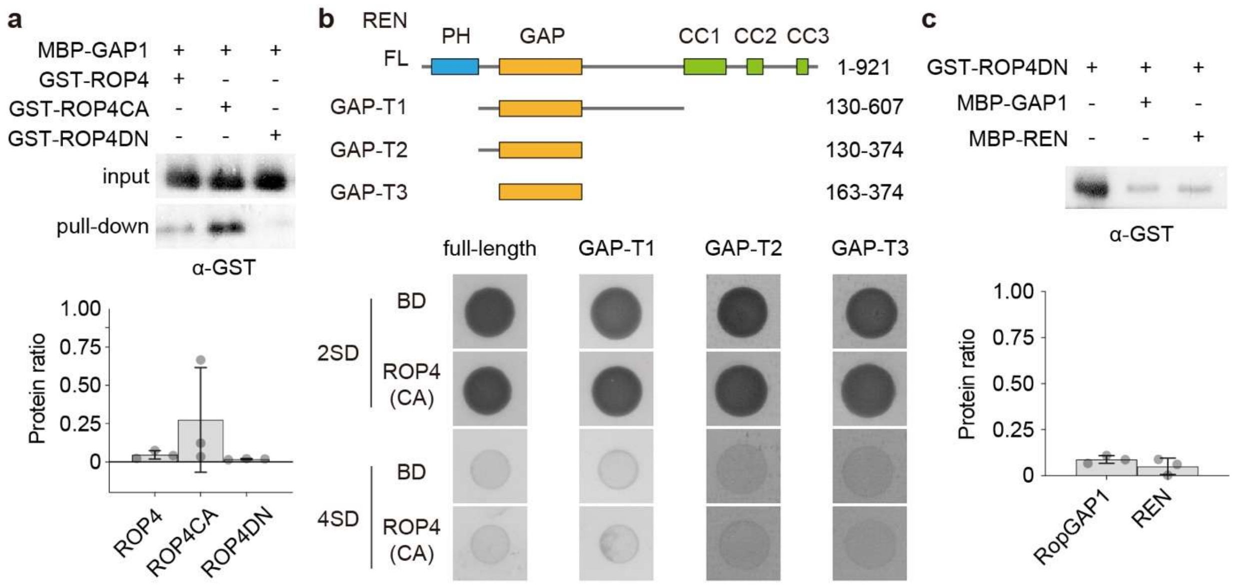

Supplementary Fig. 9: Analyses of PpRopGAP1 and PpREN interactions with different activity forms of PpROP4. (a) MBP-PpRopGAP1 preferentially interacts with the active form of PpROP4 (GST-PpROP4CA) as detected by pull-down and western blotting assays. (b) PpREN does not interact with PpROP4CA in yeast-two-hybrid assays. REN's full-length (FL) and three truncated forms (GAP-T1 to GAP-T3) were tested for interaction with PpROP4CA. PH, PLECKSTRIN HOMOLOGY domain; GAP, GTPASE-ACTIVATING domain; CC, coiled-coil motifs. (c) PpRopGAP1 and PpREN did not exhibit differences in binding capacity with the inactive form of PpROP4 (PpROP4DN). All experiments were performed with at least three replicates.

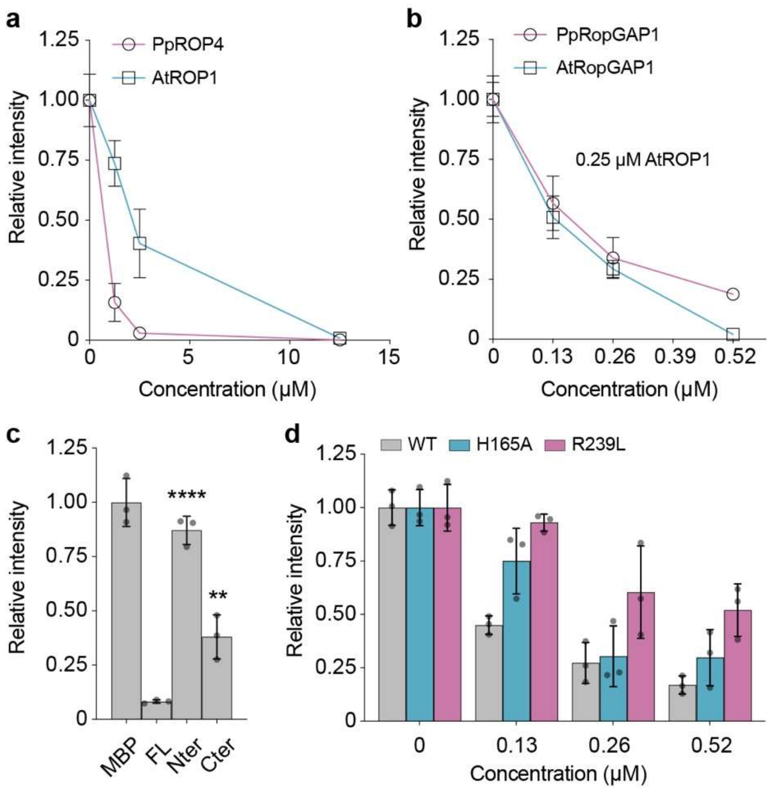

Supplementary Fig. 10: The GAP activity of wild-type and mutant forms of PpRopGAP1 in vitro. (a) The intrinsic GTPase activity of PpROP4 and AtROP1. (b) Stimulated GTPase activity of AtROP1 by PpRopGAP1 and AtRopGAP1. (c) The GAP activity of full-length (FL) and truncated forms of PpRopGAP1. Note that the Nter portion of PpRopGAP1 has little GAP activity, while its absence reduces the GAP activity of PpRopGAP1. Each group is compared with the FL PpRopGAP1 using a one-way ANOVA test. **, $p < 0.01$; ****, $p < 0.0001$. (d) The GAP activity of PpRopGAP1 carrying a mutation in the CRIB motif (H165A) or a mutation required for GAP activity (R239L). Note that H165A only reduces GAP activity at low concentrations, indicating that the CRIB motif is not essential for but enhances GAP activity. All data are derived from three replicates and are shown as mean ± SD.

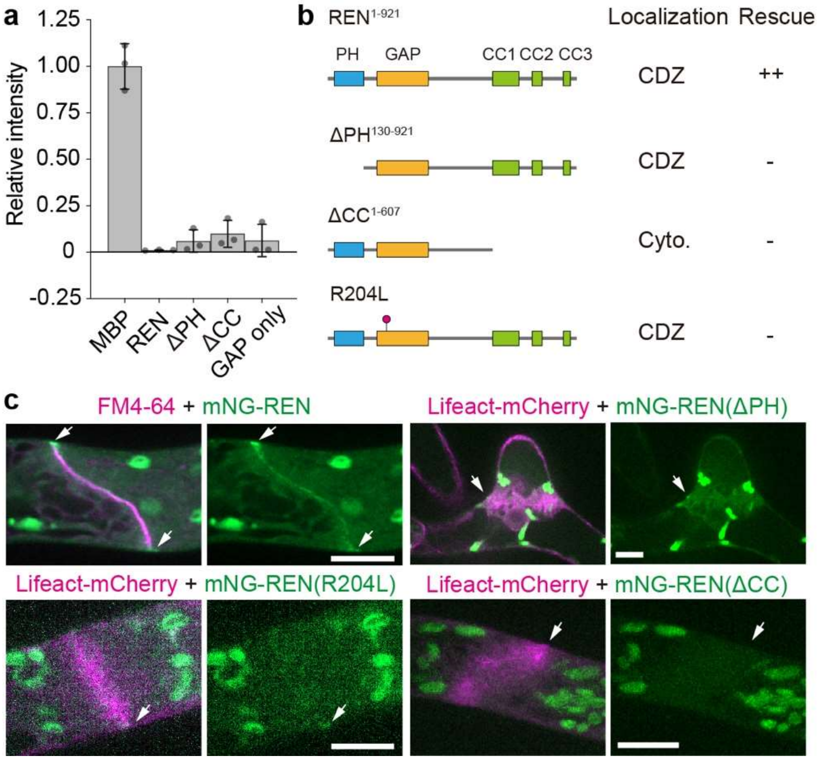

Supplementary Fig. 11: The functions of PH domain and coiled-coil motif-containing tail of PpREN. (a) The absence of PH domain (ΔPH) or coiled-coil-motif-containing tail (ΔCC) does not affect the GAP activity of PpREN. (b) Summary of localization and rescuing ability of mutant forms of PpREN. CDZ, cortical division zone. Cyto, cytoplasmic. (c) Localization of wild-type and mutant forms of PpREN at the CDZ. Arrows indicate the CDZ. Note that only the CC-motif-containing tail is essential for CDZ localization. Scale bars in all panels: 10 μm.

Supplementary Table 1. Plasmids used in this study.

Plasmid	Description	Protein Length (aa)	Protein MW [kDa]	Fusion Protein Length (aa)	Fusion Protein MW [kDa]
**Pull-down and activity analyses**					
pPY340	MBP	1-367	40.3	417	45.6
pPY376	MBP-Citrine	2-238	26.8	634	69.9
pPY287	His-PpROP4-His	2-180	19.6	228	24.7
pPY341	GST-PpROP4	2-196	21.5	433	48.7
pPY373	GST-PpROP4(G15V)	2-196	21.5	433	48.7
pPY374	GST-PpROP4(D20N)	2-196	21.5	433	48.7
pPY344	MBP-PpRopGAP1	2-469	52.0	881	96.8
pPY353	MBP-PpRopGAP1-Nter	2-193	21.1	605	65.9
pPY348	MBP-PpRopGAP1-Cter	194-469	31.0	689	75.6
pPY349	MBP-PpRopGAP1-GAP	194-380	21.0	582	63.8
pPY350	MBP-PpRopGAP1(H165A)	2-469	52.0	881	96.8
pPY351	MBP-PpRopGAP1(R239L)	2-469	52.0	881	96.8
pPY361	MBP-PpRopGAP1(R134L/R135L)	2-469	52.0	881	96.7
pPY345	MBP-PpREN	2-921	100.7	1336	145.4
pPY362	MBP-PpREN-ΔPH	163-921	82.7	1172	127.4
pPY352	MBP-PpREN-ΔCC	2-374	41.1	771	84.1
pPY355	MBP-PpREN(R204L)	2-921	100.7	1336	145.4
pPY370	MBP-PpREN(GAP domain)	163-374	23.1	603	65.7
pPY434	MBP-AtRopGAP1	2-466	51.9	878	96.6
pPY435	His-AtROP1-His	2-196	21.5	250	26.9
**Moss transgenesis**					
pPY122	PpREN knockout - NTC				
pPY123	mNG-PpREN knock-in				
pPY149	mNG-PpRopGAP1 knock-in				
pPY150	mNG-PpRopGAP2 knock-in				
pPY151	mNG-PpRopGAP3 knock-in				
pPY155	mNG-PpRopGAP4 knock-in				
pPY152	mNG-PpRopGAP5 knock-in				
pPY167	mNG-PpRopGAP6 knock-in				
pPY166	PpRopGAP1/3/4/6-sg1 - Hyg				
pPY187	PpRopGAP5-sg1 - Hyg				
pPY194	PpRopGAP2/4-sg2 - Hyg				
pPY213	PpRopGAP1-Citrine - Hyg				
pPY216	PpRopGAP1-mNG - Hyg				

pPY143	hb7-pEF1α-Cerulean - G418				
pPY140	hb7-pEF1α-Cerulean-PpREN - G418				
pPY204	hb7-pEF1α-Cerulean-PpRopGAP1 - G418				
pPY222	hb7-pEF1α-mNG-PpREN - G418				
pPY224	hb7-pEF1α-mNG-PpREN(ΔPH) - G418				
pPY225	hb7-pEF1α-mNG-PpREN(R204L) - G418				
pPY226	hb7-pEF1α-mNG-PpREN(ΔCC) - G418				
pPY229	hb7-pEF1α-mNG-PpRopGAP1 - G418				
pPY232	hb7-pEF1α-mNG-PpRopGAP1-Nter - G418				
pPY233	hb7-pEF1α-mNG-PpRopGAP1-Cter - G418				
pPY234	hb7-pEF1α-mNG-PpRopGAP1(R239L) - G418				
pPY235	hb7-pEF1α-mNG-PpRopGAP1(H165A) - G418				
pPY251	hb7-pEF1α-mNG-PpRopGAP1(S150-S193) - G418				
pPY255	hb7-pEF1α-PpRopGAP1-mNG - G418				
pPY257	hb7-pEF1α-PpRopGAP1(fs)-mNG - G418				
pPY356	hb7-pEF1α-mNG-PpRopGAP1(Q122-S193) - G418				
pPY380	hb7-pEF1α-mNG-CRIB(PpRopGAP1)-GAP(PpREN) - G418				
pPY389	hb7-pEF1α-mNG-PpRopGAP1(R134L) - G418				
pPY394	hb7-pEF1α-mNG-PpRopGAP1(R135L) - G418				
pPY412	PpROP4-swmNG knock-in				
pPY252	PpRopGEF4-mCherry - G418 knock-in				
**Yeast-two-hybrid**					
pPY274	BD-PpROP4(C193A)				
pPY275	BD-PpROP4(G15V)(C193A)				
pPY276	BD-PpROP4(T20N)(C193A)				
pPY262	AD-PpRopGAP1				
pPY267	AD-PpRopGAP1(Nter)				
pPY268	AD-PpRopGAP1(CTer)				
pPY269	AD-PpRopGAP1(H165A)				
pPY270	AD-PpRopGAP1(R239L)				
pPY293	AD-PpROPGAP1-Cter(R239L)				
pPY385	AD-PpRopGAP1(R134L)				
pPY386	AD-PpRopGAP1(R135L)				
pPY357	AD-PpRopGAP1(R134/135L)				
pPY400	AD-PpRopGAP1-Nter(R134L)				
pPY401	AD-PpRopGAP1-Nter(R135L)				
pPY406	AD-PpRopGAP1(Q122-S193)				
pPY410	AD-PpRopGAP(S150-S193)				
pPY414	AD-PpRopGAP(S150-S193, H165A)				
pPY263	AD-PpREN				

pPY271	AD-PpREN(ΔPH)				
pPY272	AD-PpREN(ΔCC)				
pPY283	AD-PpREN(130-607)				
pPY478	AD-PpREN(130-374)				
pPY511	AD-PpREN(163-374)				
