# Supplementary figures and images for: Two subtypes of GTPase-activating proteins coordinate tip growth and cell size regulation in Physcomitrium patens
Source: Nat Commun. 2023 Nov 4;14:7084. doi: 10.1038/s41467-023-42879-y (PMC10625565; doi:10.1038/s41467-023-42879-y)

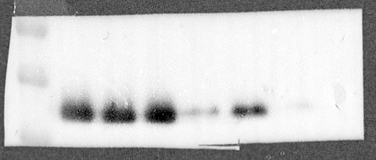

Supplement: Supplementary file 13 — Source Data [file 41467_2023_42879_MOESM13_ESM.zip › Source Data/Supplementary_Figure_9a_uncropped.tif]

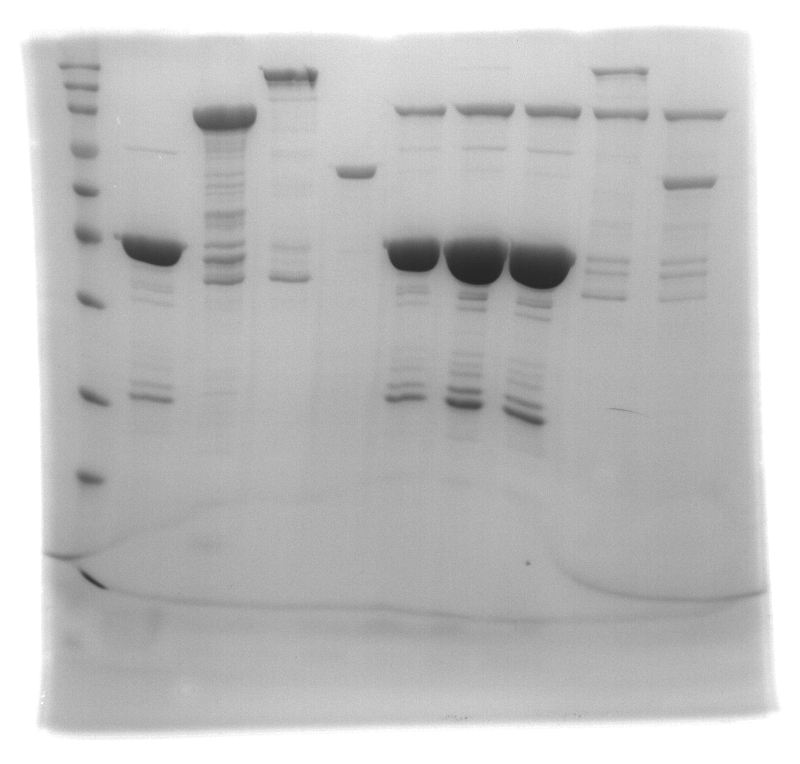

Supplement: Supplementary file 13 — Source Data [file 41467_2023_42879_MOESM13_ESM.zip › Source Data/Figure_9a_uncropped.tif]

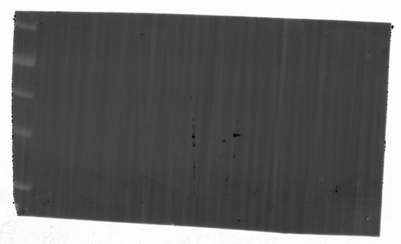

Supplement: Supplementary file 13 — Source Data [file 41467_2023_42879_MOESM13_ESM.zip › Source Data/Figure_8e_uncropped.tif]

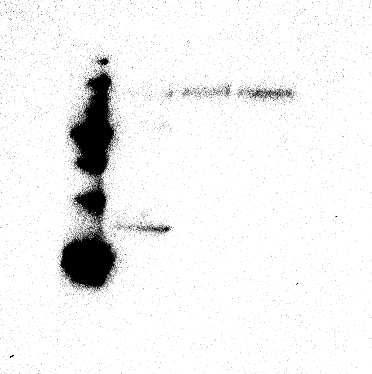

Supplement: Supplementary file 13 — Source Data [file 41467_2023_42879_MOESM13_ESM.zip › Source Data/Figure_3d_uncropped_top.tif]

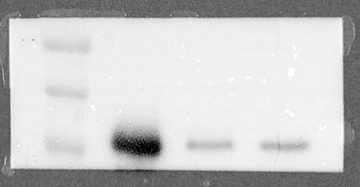

Supplement: Supplementary file 13 — Source Data [file 41467_2023_42879_MOESM13_ESM.zip › Source Data/Supplementary_Figure_9c_uncropped.tif]

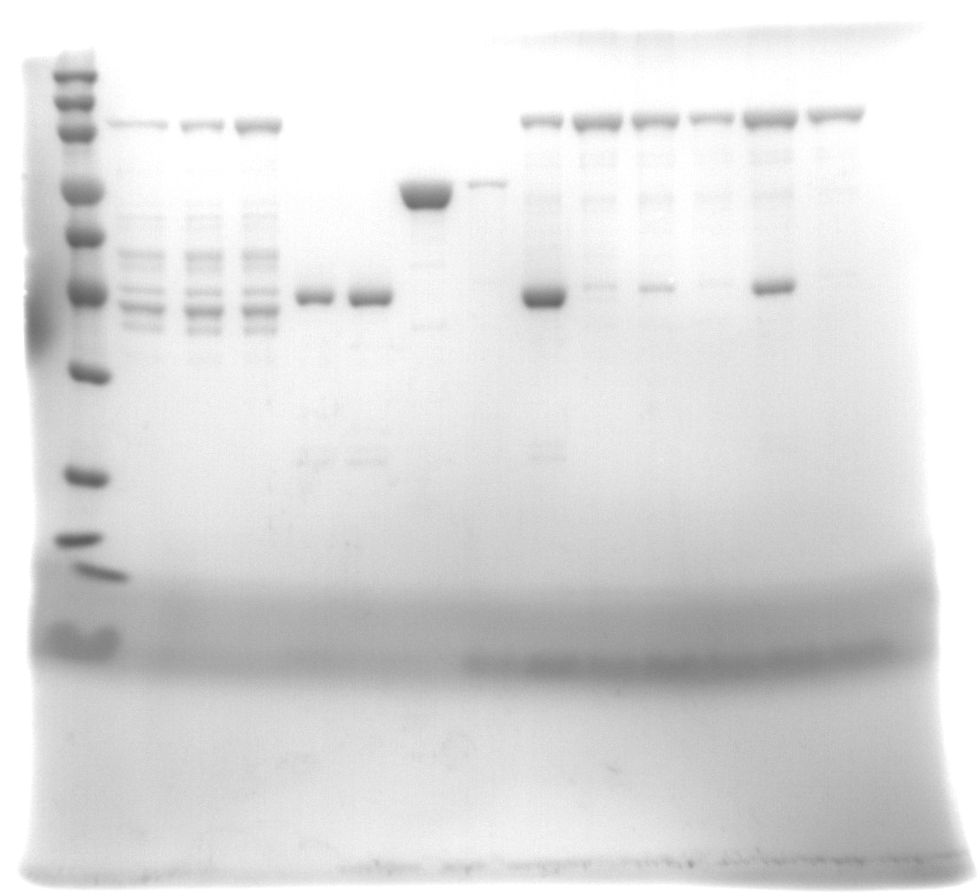

Supplement: Supplementary file 13 — Source Data [file 41467_2023_42879_MOESM13_ESM.zip › Source Data/Figure_8c_uncropped.tif]

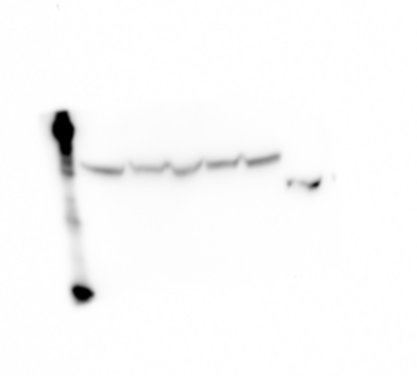

Supplement: Supplementary file 13 — Source Data [file 41467_2023_42879_MOESM13_ESM.zip › Source Data/Figure_3d_uncropped_bottom.tif]
